# TNF ROCKs the boat as the kidney endothelium springs a leak

**DOI:** 10.14814/phy2.12678

**Published:** 2016-01-12

**Authors:** Roderick J. Tan

**Affiliations:** ^1^ Department of MedicineUniversity of Pittsburgh School of MedicinePittsburghPennsylvania

Abnormal vascular permeability is a hallmark of sepsis‐mediated injury to various organs. An intact endothelial barrier is critical to homeostasis and prevents leakage of abnormal quantities of fluid and protein, including proinflammatory cytokines and chemokines, into the extravascular space. Barrier disruption leads to distributive shock as well as organ dysfunction and edema (Lee and Slutsky 2010).

Extensive research implicates tumor necrosis factor‐*α* (TNF) in this process. TNF binds receptors on the endothelial cell surface and activates intracellular Rho GTPases. This increases Rho‐associated kinase (ROCK) activity which inactivates myosin light chain (MLC) phosphatase and, in the presence of myosin light chain kinase (MLCK), leads to MLC phosphorylation. As a result, actin filaments reorganize into stress fibers, and cells exhibit increased actomyosin contractility, leading to endothelial barrier disruption (Marcos‐Ramiro et al. 2014).

Endothelial permeability in the kidney has unique manifestations. Similar to other organs, dysfunction of endothelia in the peritubular capillaries leads to reduced blood flow and inflammation that contribute to AKI pathogenesis (Molitoris 2014). In addition, the specialized glomerular endothelium, together with the glomerular epithelium (i.e., podocytes) and associated basement membrane, forms the kidney's filtration barrier. Disruption of the filtration barrier leads to proteinuria, or the abnormal accumulation of protein in the urine, and this is a characteristic of many progressive kidney diseases. While much research has focused on the podocyte in proteinuria, increasing evidence supports a role for the endothelium as well. The structure of glomerular endothelium is also unusual, including fenestrae that lack the diaphragms present elsewhere (Satchell and Braet 2009). These differences suggest the need for additional studies specifically on renal endothelium. This knowledge gap has been a focus of Cunningham and colleagues for many years, and they build on previous work in their current publication.

Xu et al. examine TNF‐mediated renal endothelial dysfunction utilizing both immortalized human glomerular endothelial cell cultures (GeNCs) and isolated primary mouse renal endothelial cultures (ReNCs) (Xu et al. 2015). The authors first demonstrate that LPS exposure in mice leads to a rapid and significant loss of barrier integrity, with leakage of plasma contents into the renal interstitium within 24 h of exposure. Exposure of GEnCs and REnCs to TNF also increased permeability and actin filament reorganization in a ROCK‐ and MLCK‐dependent manner. Although tight junctions and caspases were previously shown to be affected by endotoxemia and TNF (Wu et al. 2009; Eadon et al. 2012), examination of tight junctions here revealed only minor alterations in occludin and ZO‐1, and caspase inhibition did not affect permeability.

The authors previously determined that TNF leads to glycocalyx degradation and also alters both the size and number of fenestrae in kidney endothelium (Xu et al. 2014). Here, they confirm glycocalyx disruption in TNF‐exposed GEnCs that was ameliorated with ROCK and MLCK inhibition. In addition, TNF increased fenestrae number while decreasing their average diameter, and these changes were dependent on MLCK. Cumulatively, these and previous findings support an important role for the TNF–ROCK–MLCK pathway in renal endothelial function via actin restructuring, glycocalyx degradation, and changes in fenestral structure.

A number of future directions are suggested by these intriguing data. Although technically challenging, the validation of these experimental findings in human AKI is paramount. Similarly, while these studies were facilitated by homogeneous cell cultures, interactions with the local microenvironment likely play a significant role. As examples, the authors previously described increases in the glycocalyx‐degrading enzyme heparanase in vivo after TNF exposure but could not demonstrate this in vitro in this study. Further, the fenestrae changes in GEnCs exposed to TNF are exactly opposite to what they observed in TNF‐injected mice, in which there was a decrease in number with an increase in size (Xu et al. 2014). These differences may be inherent to cell cultures lacking interactions with associated epithelium, inflammatory cells, and extracellular matrix and suggest the need for more complex biological models.

The mechanisms leading to the glycocalyx and fenestrae changes are also unknown, as are their exact roles in pathology. Since glycocalyx degradation was not due to increased heparanase, other proteases or factors must be responsible. Furthermore, while decreased glycocalyx may increase permeability in the glomerulus and lead to proteinuria, loss of glycocalyx in peritubular capillaries may have other roles, such as effects on leukocyte adhesion in sepsis‐induced AKI. In the fenestrae, while MLCK inhibition abrogated changes in size and number, ROCK inhibition only affected the number, suggesting divergent pathways for controlling size. Further interrogation of these pathways is required, and the actual overall importance of fenestral changes on glomerular permeability requires further study.

Nonetheless, the findings of this article are significant on several fronts. First, the effects of TNF specifically on renal endothelium requires close study, since sepsis‐related AKI involves increased endothelial permeability (Molitoris 2014). Second, although proteinuria is not a strong characteristic of AKI, its presence in septic AKI can predict worsened outcomes or serve as a biomarker for systemic endothelial permeability (Gosling et al. 2006). Third, study of endothelial dysfunction is critical to understand the observation of microvascular peritubular dropout or rarefaction after episodes of AKI, a phenomenon that predisposes to further AKI and permanent renal failure (Basile et al. 2001; Horbelt et al. 2007). Fourth, it validates the role of TNF, ROCK, and MLCK on not only the renal endothelial cell cytoskeleton but the structure of the associated glycocalyx and fenestrae. The fenestrae changes are particularly notable because they are difficult to study and require the unbiased ultrastructural analysis described in this article.

Finally, it should be noted that these findings may be applicable not only for sepsis‐induced AKI but for other kidney diseases characterized by significant proteinuria. Elevated TNF levels have been linked to a number of kidney diseases including diabetic nephropathy (Awad et al. 2015). It will be interesting to determine whether inhibition of the TNF–ROCK–MLCK pathway can reduce proteinuria and improve outcomes in these diseases. As such, greater understanding of renal endothelial biology may lead to improved treatment of a much broader patient population for whom we have limited options.
